# Solasonine, A Natural Glycoalkaloid Compound, Inhibits Gli-Mediated Transcriptional Activity

**DOI:** 10.3390/molecules21101364

**Published:** 2016-10-14

**Authors:** Jun Yang, Wenjing Huang, Wenfu Tan

**Affiliations:** Department of Pharmacology, School of Pharmacy, Fudan University, 826 Zhangheng Road, Shanghai 201203, China; 14211030042@fudan.edu.cn (J.Y.); 15211030052@fudan.edu.cn (W.H.)

**Keywords:** solasonine, hedgehog, Gli resistance

## Abstract

The major obstacle limiting the efficacy of current Smoothened (Smo) inhibitors is the primary and acquired resistance mainly caused by Smo mutations and Gli amplification. In this context, developing Hh inhibitors targeting Gli, the final effector of this signaling pathway, may combat the resistance. In this study we found that solasonine, a natural glycoalkaloid compound, significantly inhibited the hedgehog (Hh) pathway activity. Meanwhile, solasonine may obviously inhibit the alkaline phosphatase (ALP) activity in C3H10T1/2 cells, concomitantly with reductions of the mRNA expression of *Gli1* and *Ptch1*. However, we found that solasonine exhibited no effect on the transcriptional factors activities provoked by TNF-α and PGE2, thus suggesting its selectivity against Hh pathway activity. Furthermore, we identified that solasonine inhibited the Hh pathway activity by acting on its transcriptional factor Gli using a series of complementary data. We also observed that solasonine obviously inhibited the Gli-luciferase activity provoked by ectopic expression of Smo mutants which may cause the resistance to the current Smo inhibitors. Our study suggests that solasonine may significantly inhibit the Hh pathway activity by acting on Gli, therefore indicating the possibility to use solasonine as a lead compound to develop anticancer drugs for combating the resistance of current Smo inhibitors.

## 1. Introduction

The Hedgehog (Hh) signaling pathway, an evolutionarily conserved signaling axis, plays critical roles in various physiological and pathological conditions, such as embryonic patterning, tissue homeostasis, and cancer [1,2]. Three Hh ligands, including Sonic Hh (Shh), Indian Hh (Ihh), Desert Hh (Dhh), have been identified so far. The binding of Hh ligands to ptch, a 12-transmembrane cell surface receptor, may relieve the inhibitory effect of ptch on Smoothened (Smo), a member of the 7-transmemebrane cell surface receptors. This consequently allows the accumulation of Smo in the primary cilium. Ultimately, canonical Hh signaling regulates the activity, proteolytic processing, and the stability of the Gli family transcriptional factors, Gli1-3, and subsequently initiates the transcription of Gli-dependent target genes, such as *Gli1* and *ptch1*. This regulation involves a number of protein kinases, including protein kinase A (PKA), glycogen synthase kinase 3 (GSK3) and case kinase 1, and the negative regulator suppressor of fused (SuFu) [2].

Aberrant activation of Hh signaling pathway has been well-documented to be required for the growth of various types of cancers, such as basal cell carcinoma (BCC), medulloblastoma, colorectal cancer, and small lung cell cancer, to name but a few [3]. This has led to large scale of small molecular inhibitors developed for treatment of cancers dependent on the Hh pathway. The vast majority of current Hh inhibitors function by targeting Smo. Among these Smo inhibitors, GDC-0449 and LDE-225 have successively been approved for clinic treatment of advanced BCC with aberrant Hh activity due to loss of the functional allele of ptch [4].

Considering that both GDC-0449 and LDE-225 inhibit the Hh pathway activity by targeting Smo, it is not surprising that they are primarily insensitive for cancers harboring aberrant Hh activity caused by genetic alterations in key components downstream of ptch, such as activating mutations in Smo, loss of Sufu, or Gli2 gene amplification [5,6]. Moreover, like other mechanism-based anticancer drugs, after an initial rapid regression, the cancers frequently evolve resistance to Smo inhibitors. Multiple mechanisms have been shown to be responsible for this evasion of Smo inhibition, such as secondary mutations in Smo, loss of Sufu, and Gli2 amplification [5,6]. Due to the molecular basics underlying the primary and acquired resistance to current Smo inhibitors, it is conceivable that targeting Gli, the final effector of the Hh signaling pathway, may combat the primary and acquired resistance of current Smo inhibitors [7]. Hence, there remains a critical need to develop Hh inhibitors acting on Gli.

**Figure 1:**
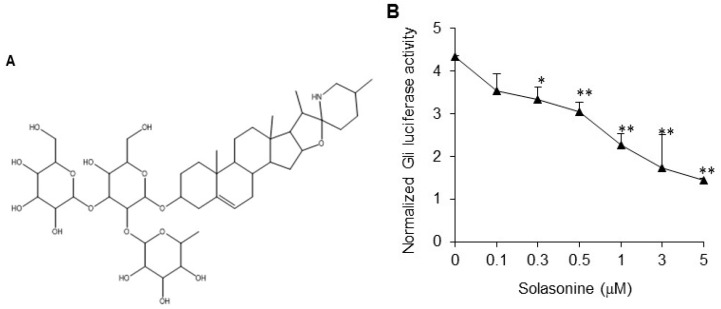

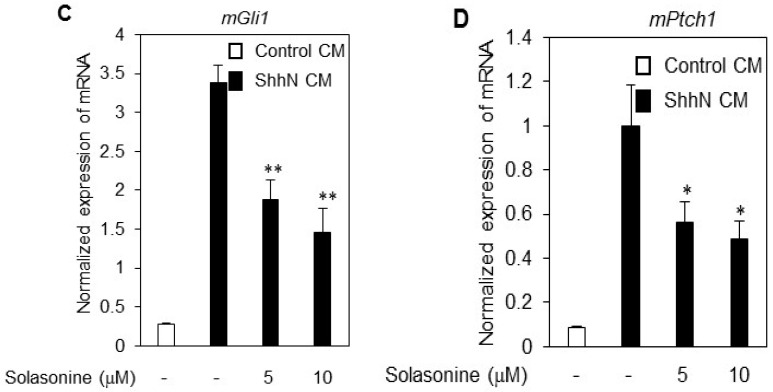
Solasonine inhibits Hh pathway activity in light II cells. (**A**) Chemical structure of solasonine. (**B**) Solasonine inhibited the Gli-luciferase activity in response to ShhN CM. Light II cells seeded in 96-well plates were exposed to ShhN CM supplemented with various concentrations of solasonine for 36 h. The cells were collected for dual luciferase reporter assys. The firefly luciferase activity was normalized to the *Renilla*-luciferase activity. Data are expressed as mean ± s.d. (*n* = 3); (**C**,**D**) Solasonine inhibited the mRNA expression of *Gli1* (**B**) and *Ptch1* (**C**) stimulated by ShhN CM. Light II cells treated with ShhN CM containing various concentrations of solasonine for 24 h were harvested and subjected to qRT-PCR analysis. Results are expressed as mean ± s.d. (*n* = 3). Statistical differences were analyzed by the two-tailed Student′s *t* test and *p* < 0.05 was considered as significant (* *p* < 0.05; ** *p* < 0.01).

Glycoalkaloids are secondary plant metabolites found in a wide range of Solanaceous plants, for example eggplants, potatoes, and tomatoes. Numerous glycoalkaloids have been isolated from these plants, such as solamargine, solasonine, chaconine, solanine, and tomatine, to name but a few [8]. When screening for Hh inhibitors from natural compounds, we observed that the natural compound solasonine obviously inhibited the Gli-luciferase activity provoked by Shh. Herein, we report that solasonine, with the chemical structure shown in Figure 1A, may selectively inhibit the Hh signaling pathway activity by targeting Gli, and exhibits potential for combating the primary and acquired resistance to current Smo inhibitors.

## 2. Results

### 2.1. Solasonine Inhibits Hh Signaling in Light II Cells

We used light II cells, NIH-3T3 cells stably transfected with a Gli-responsive firefly luciferase reporter and *Renilla*-luciferase expression vector, to examine the inhibitory effect of solasonine (Figure 1A) on the Hh signaling activity stimulated by condioned medium made by transfection of ShhN, a plasmid containing the *N*-terminal signaling domain of the Shh. We observed that solasonine obviously inhibited the Gli-responsive reporter activity stimulated by ShhN CM in a dose-dependent manner (Figure 1B), with an IC_50_ value of 1.56 μM. Simultaneously, solasonine suppressed the mRNA expression of *Gli1* (Figure 1C) and *Ptch1* (Figure 1D), two transcriptional targets of Gli, which frequently served as readouts of Gli activity [3]. These results suggest that solasonine may be an Hh inhibitor.

### 2.2. Solasonine Inhibits the Alkaline Phosphatase (ALP) Activity in C3H10T1/2 Cells

The mouse embryonic fibroblast line C3H10T1/2 is a mesenchymal stem cell line that can differentiate into adipocytes, chondrocytes, and bone osteoblasts [9,10]. The Hh signaling pathway may promote the differentiation of C3H10T1/2 cells into the bone cell lineage and ALP induction has been used as a marker for this process [11]. To further confirm the inhibitory effect of solasonine on the Hh signaling activity, we assessed the influence of solasonine on the ALP activity in C3H10T1/2 cells.

**Figure 2:**
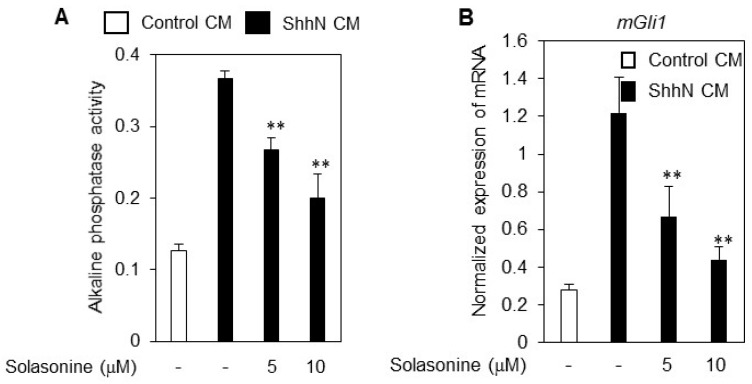

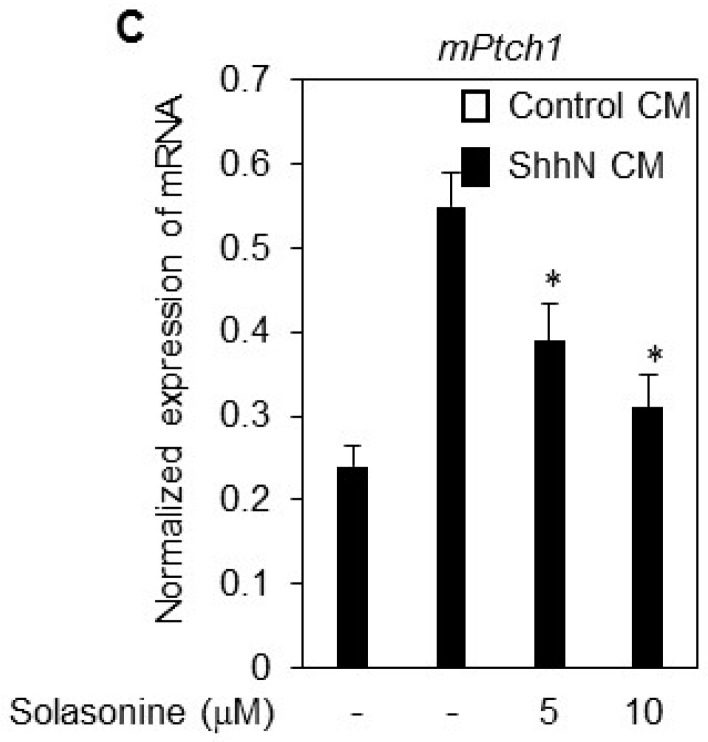
Solasonine inhibits Hh pathway activity in C3H10T1/2 cells. (**A**) Solasonine suppressed the ALP activity in response to ShhN CM in C3H10T1/2 cells. Data are expressed as mean ± s.d.; (**B**,**C**) Solasonine inhibited the mRNA expression of *Gli1* (**B**) and *Ptch1* (**C**) provoked by ShhN CM in C3H10T1/2 cells. After treated for 36 h with ShhN CM with or without distinct concentrations of solasonine, C3H10T1/2 cells were harvested for qRT-PCR analysis. Results are expressed as mean ± s.d. (*n* = 3). Statistical differences were analyzed by the two-tailed Student′s *t* test and *p* < 0.05 was considered as significant (* *p* < 0.05; ** *p* < 0.01).

As shown in Figure 2A, treatment with solasonine obviously suppressed the ALP activity in C3H10T1/2 cells. Meanwhile, exposure of C3H10T1/2 cells to solasonine caused reduction of the mRNA expression of *Gli1* (Figure 2B) and *Ptch1* (Figure 2C), further supporting the argument that solasonine may inhibit the Hh activity.

### 2.3. Solasonine Displays Selectivity for Inhibiting Hh Pathway Activity

**Figure 3:**
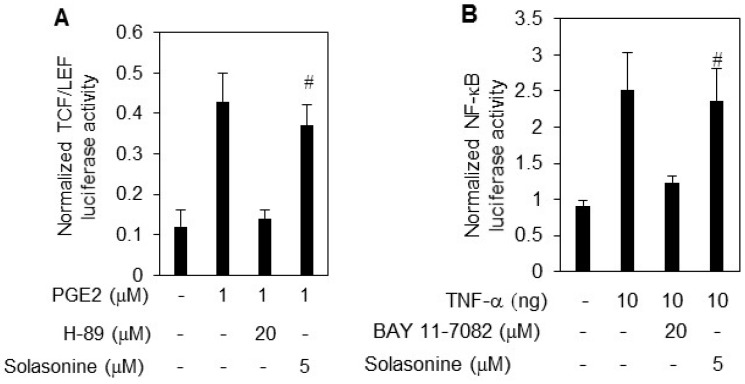
Solasonine possesses selectivity for inhibiting Hh pathway activity. (**A**) Solasonine had no effect on the NF-κB activity. The 293T cells transfected with luciferase reporter plasmids with NF-κB binding sites and TK-*Renilla* plasmids were treated with TNF-α with or without BAY 11-872 or solasonine for 24 h. The results are expressed as mean ± s.d. (*n* = 3); (**B**) Solasonine had no effect on the TCF/LEF activity. The LS174T cells were transfected with luciferase reporter constructs with TCF/LEF binding sites and the TK-*Renilla* plasmids, and subjected to PGE2 with various concentrations of solasonine or H89 for 24 h. The results are expressed as mean ± s.d. (*n* = 3). Statistical differences were analyzed by the two-tailed Student′s *t* test and *p* < 0.05 was considered as significant # *p* > 0.05.

To rule out the possibility that solasonine nonspecifically inhibits Hh activity provoked by ShhN CM, we next examined the effect of solasonine on the activity of other transcriptional factors, such as NF-κB, and TCF/LEF [12]. The results showed that TNF-α and PGE2 obviously stimulated the NF-κB (Figure 3A), and TCF/LEF luciferase activity (Figure 3B). However, we observed that solasonine failed to exhibit inhibitory activity against either NF-κB (Figure 3A), or TCF/LEF luciferase activity (Figure 3B). The BAY 11-7082, and H89 were used as positives for inhibition of NF-κB (Figure 3A), and TCF/LEF luciferase activity (Figure 3B), respectively.

To further confirm the selectivity of the inhibitory effect of solasonine on Hh pathway activity, we tested its effect on a panel of kinases activity, especially the receptor tyrosine kinases that have been the most frequently used targets to develop molecular targeted anti-cancer drugs, such as vascular growth factor receptor 1 (VEGFR1), VEGFR2, c-kit, RET, three members of epidermal growth factors (EGFR) family, ephrin type-A receptor 2 (EPH-A2), insulin-like growth factor 1 receptor (IGF1R), fibroblast growth factor receptor (FGFR1). We found that solasonine had little effect on those kinase activity (data not shown). Hence, these data demonstrate that solasonine possess selectivity when suppressing Hh signaling pathway activity.

### 2.4. Solasonine Inhibits the Hh Signaling Pathway by Targeting Gli

**Figure 4:**
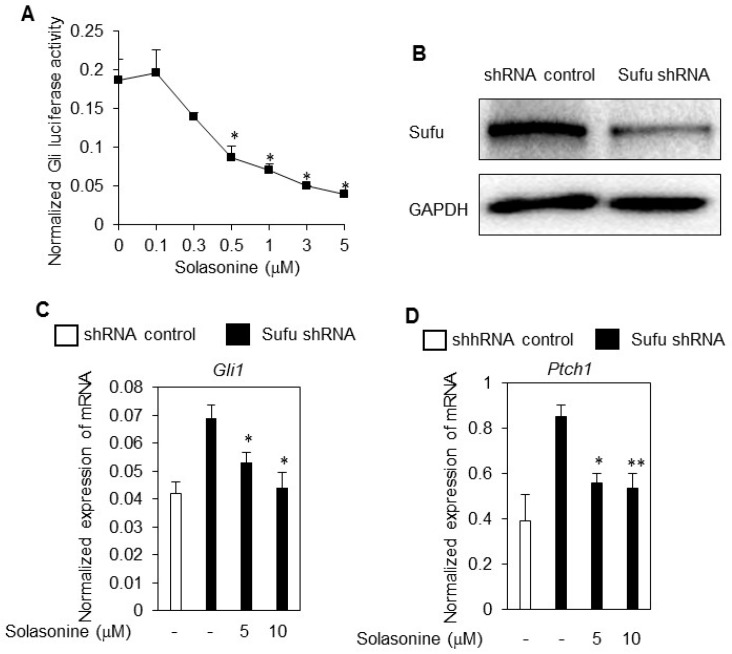

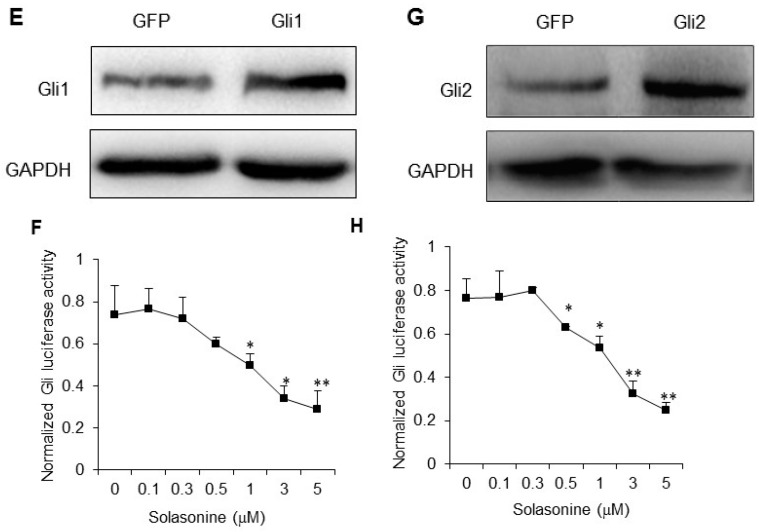
Solasonine inhibits the Hh signaling pathway by targeting Gli (**A**) Solasonine dose-dependently inhibits the Gli-luciferase activity provoked by SAG. The results are expressed as mean ± s.d.; (**B**). Expression of Sufu in NIH-3T3 cells infected with lentivirus carrying Sufu shRNA or shRNA control. The cells infected with various types of lentivirus were collected for Western blot analyses. Immunoblotting of GAPDH served as a loading control; (**C**,**D**) Solasonine suppressed the mRNA expression of Gli1 (**C**) and ptch1 (**D**) caused by limiting Sufu expression. The results are expressed as mean ± s.d.; (**E**) Ectopic expression of Gli1 in light II cells as examined by western blot analysis; (**F**) Solasonine suppressed the Gli-luciferase activity caused by exogenous expression Gli1 in a dose-dependent manner. Data are expressed as mean ± s.d. (*n* = 3); (**G**) Ectopic expression of Gli2 in light II cells as examined by western blot analysis; (**H**) Solasonine suppressed the Gli-luciferase activity caused by exogenous expression Gli2 in a dose-dependent manner. Data are expressed as mean ± s.d. (*n* = 3). Statistical differences were analyzed by the two-tailed Student′s *t* test and *p* < 0.05 was considered as significant (* *p* < 0.05; ** *p* < 0.01).

Having demonstrated that solasonine may selectively inhibits the Hh signaling pathway activity, we then set out to define the molecular target of solasonine for inhibiting Hh pathway activity. First, we assessed the influence of solasonine on the Gli-luciferase activity provoked by SAG, a specific small molecule agonist of Smo [13]. As shown in Figure 4A, we observed that solasonine dose-dependently inhibited the Gli-luciferase activity in response to SAG with an IC_50_ value of 1.43 μM, similar to that for inhibiting the Gli-luciferase activity provoked by ShhN CM. These results suggest that solasonine inhibits the Hh pathway activity by targeting molecules downstream of Smo, or by acting on Smo with a distinct binding site from that of SAG [13]. We next determine the effect of solasonine on the Hh activity provoked by limiting the expression of Sufu, a negative regulator of Hh pathway [3]. The results showed that solasonine significantly suppressed the Hh pathway activity caused by Sufu knockdown via lentivirus mediated Sufu shRNA in NIH-3T3 cells (Figure 4B), as reflected by decreasing the mRNA expressions of Gli1 (Figure 4C) and ptch1 (Figure 4D).

These results suggest that solasonine suppress the Hh pathway by targeting molecules downstream of Sufu. We, therefore, asked whether solasonine may inhibit the Gli-luciferase activity stimulated by Gli, the transcriptional factor of Hh pathway. We observed that solasonine obviously inhibited the Gli-luciferase activity provoked by ectopic expression of Gli1 (Figure 4E,F) or Gli2 (Figure 4G,H) in light II cells in a dose dependent manner. The IC_50_ values of solasonine inhibiting the Gli-luciferase activity provoked by artificially ectopic expression of Gli1 or Gli2 were 2.49 μM, and 2.30 μM, respectively, similar to those for inhibiting the Hh pathway activity in response to ShhN CM or SAG (*p* values of statistical analysis are 0.329, and 0.153, respectively). Collectively, our data clearly demonstrate that solasonine inhibits the Hh pathway activity by targeting the transcriptional Gli, thus suggesting its potential effect on combating current Smo inhibitors resistance caused by Gli amplification.

### 2.5. Solasonine Inhibits the Hh Pathway Activity Provoked by Distinct Smo Mutants

**Figure 5:**
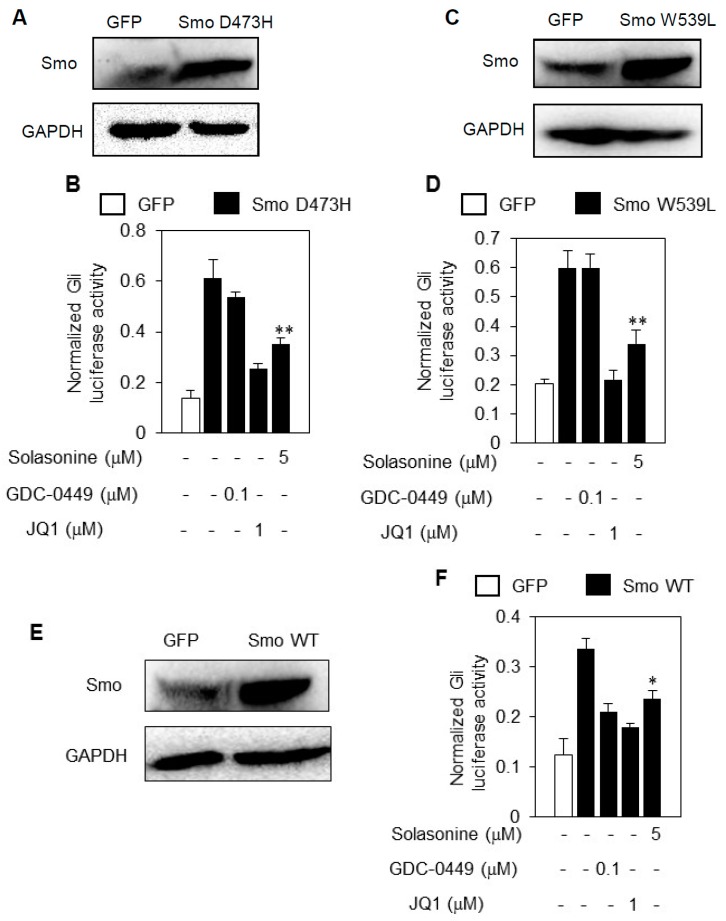
Solasonine inhibits the Hh pathway activity provoked by ectopic expressions of distinct Smo plasmids. (**A**) Ectopic expression of SmoD473H in light II cells as examined by western blot analysis; (**B**) Solasonine suppressed the Gli-luciferase activity provoked by exogenous SmoD473H. Light II cells transfected with GFP and SmoD473H plasmids were exposed to various treatments as indicated for 36 h. Data are expressed as mean ± s.d. (*n* = 3); (**C**) Ectopic expression of SmoW539L in light II cells as examined by western blot analysis; (**D**) Solasonine suppressed the Gli-luciferase activity provoked by artificially forced expression of SmoW539L. Light II cells transfected with GFP and SmoW539L plasmids were subjected to various treatments as indicated for 36 h. Data are expressed as mean ± s.d. (*n* = 3); (**E**) Ectopic expression of Smo wild type in light II cells as examined by western blot analysis; (**F**) Solasonine blocked the Gli-luciferase activity stimulated by artificially forced expression of Smo wild type. Light II cells transfected with GFP and Smo wild type plasmids were subjected to various treatments as indicated for 36 h. Data are expressed as mean ± s.d. (*n* = 3). Statistical differences were analyzed by the two-tailed Student′s *t* test and *p* < 0.05 was considered as significant (* *p* < 0.05; ** *p* < 0.01).

One of major molecular mechanisms underlying the primary and acquired resistance of current Smo inhibitors is gain-of-function mutations in their target Smo [14]. Having characterized Gli as the cellular target of solasonine, we then proceeded to determine whether solasonine may inhibit the aberrant Hh pathway activity caused by ectopic expression of Smo mutants SmoD473H (Figure 5A) or SmoW539L (Figure 5C), which both may cause resistance to the current Smo antagonists [5,6]. The results showed that treatment with solasonine robustly suppressed the Gli-luciferase activity provoked by artificially forced expression of Smo D473H (Figure 5B) or Smo W539L (Figure 5D) into light II cells, as does JQ1, a specific small molecular inhibitor of Gli [15].

However, GDC-0449, a Smo inhibitor approved for clinic treatment of advanced BCC [16], failed affect the Gli-luciferase activity caused by either Smo D473H (Figure 5B) or Smo W539L (Figure 5D). Moreover, solasonine, GDC-0449, and JQ1 all repressed the Gli-luciferase activity caused by ectopic expression of wild type Smo (Figure 5E,F). Hence, our data suggest that solasonine possess potential for overcoming the current Smo inhibitors resistance caused by Smo mutations.

## 3. Discussion

In this study, we have shown that solasonine may inhibit the Hh signaling pathway by acting on Gli. Meanwhile, we observed that solasonine possessed selectivity against inhibiting Hh pathway activity, as supported by its minimal effect on the transcriptional activity provoked by PGE2 or TNF-α. Our data that solasonine is an Hh inhibitor by targeting Gli are consistent with the report from Mayank, who predicted solasonine as a Gli inhibitor by molecular docking method [17]. Solasonine is a major glycoalkaloid found in eggplant (*Solanum melongena*) and in a wide range of other *Solanum* species [18]. Previous study from other laboratory has shown that solasonine possess cytotoxic activity against a panel of tumor cell lines [19]. Our present study reveals a novel molecular mechanism behind the anticancer action of solasonine.

Adverse effects of steroidal glycoalkaloids are manifested in a number of ways, such as reduced respiratory activity or blood pressure, bradycardia and haemolysis, likely due to membrane disruption, inhibition of acetylcholinesterase activity [20]. Using frog embryo as a model, Blankemeyer et al reported that solasonine caused disruption of membrane potential of albino *Xenopus* embryos with EC_50_ of 21 μM, and mortality with LC_50_ of 6.03, 8.21 μM for solasonine buffered at pH 6, and 8, respectively [18]. Although the IC_50_ value (1.56 μM) for solasonine inhibiting the Gli-luciferase activity provoked by Shh CM is lower than EC_50_ and LC_50_ values of adverse effect obtained with frog embryo, much attention should be paid when translating the inhibitory effect of solasonine on Hh pathway into human settings. Generally, our study shows that solasonine may inhibit Hh pathway by targeting Gli, and thus suggesting it may be a good lead compound for developing more potent and safety Gli inhibitors.

The primary and acquired resistance largely handle the clinical efficacy of current Smo inhibitors. Given the underlying molecular mechanisms responsible for the Smo inhibitors resistance, which frequently caused by genetic alterations in molecules downstream of ptch [5,6], particular interest has been focused on developing inhibitors able to target Gli, the final effector of the Hh pathway, to combat the resistance of Smo inhibitors [7]. The present study clearly demonstrated that solasonine is an Hh inhibitor by targeting Gli. Moreover, our data showed that solasonine obviously suppressed the Gli-lucifearse activity provoked by ectopic expression of Smo mutants and Gli. Hence, our study suggests that solasonine possesses potential to combat Smo inhibitors resistance, and suggests solasonine is a good candidate for developing Hh inhibitors to combat the resistance of current Smo inhibitors. Due to the loss of Hh activity of the long term cultured tumor cell lines [21], this study lacks data of the in vitro and in vivo anti-cancer effect of solasonine. Hence, further evaluation using Hh driven cancer model made by genetic engineering modified mice [22] is critical for completely understanding the effect of solasonine on inhibiting the growth of Hh driven cancer, as well as combating the resistance of current Smo inhibitors.

A wide range of Hh inhibitors have been developed to inhibit the Hh pathway activity by targeting Gli. According to their molecular mechanisms, these Gli inhibitors have been clarified as direct and indirect Gli inhibitors [7]. The direct Gli inhibitors, such as GANT-61 [23], GANT-58 [23], and As_2_O_3_ [24], et al., usually inhibit the Hh pathway activity by directly binding the transcriptional factor Gli, consequently interfering its transcriptional ability. On other hand, indirect Gli inhibitors function by acting on the molecules critical for Gli, and consequently affecting its proteolytic degradation or post-translational modifications. These indirect Gli inhibitors include JQ1 [15], pyrvinium [25], CMAPs [26], imiquimond [27], to name a few. As we have characterized solasonine as a Gli inhibitor in this study, therefore, how solasonine inhibits the Hh pathway activity by targeting Gli warrants further investigations.

## 4. Materials and Methods

### 4.1. Reagents, Cell Lines and Cell Culture

Solasonine was purchased from Yuanye Biotech. (Jinan, China). Prostaglandin E2 (PGE2) were obtained from Sigma-Aldrich (St. Louis, MO, USA). The Hh pathway antagonists GDC-0449 and JQ1 were obtained from Selleckchemicals (Houston, TX, USA). The tumor necrosis factor α (TNF-α), BAY 11-8072 and H89 were purchased from Beyotime (Suzhou, China). Plasmids used in this study were described as previously reported [28]. The cells used in this study, including light II cells, NIH-3T3 and C3H/10T1/2 mouse embryo fibroblast cells, HEK-293T human epithelial kidney cells, and LS174T colon cancer cells, were all obtained from the American Type Culture Collection (Manassas, VA, USA), and were routinely cultured according to the manufacturer′s instructions. HEK-293T were transfected with ShhN and GFP plasmids. After transfection of 48 h the ShhN conditioned medium (ShhN CM) and GFP CM were collected as previously described [28].

### 4.2. Dual Luciferase Assays

Cells transfected with luciferase reporter plasmids containing respective binding-sites of various transcriptional factors and Renilla-TK construct (Promega, Madison, WI, USA) were seeded into 48-well plates. After various treatments as indicated, luciferase assays were conducted using a dual luciferase assay kit according to the manufacturer′s instructions (Promega) on a luminometer (Molecular Devices, Sunnyvale, CA, USA). The firefly luciferase values were normalized to *Renilla* values.

### 4.3. Alkaline Phosphatase Activity Assay

C3H10T1/2 cells were plated into 96-well plates at a density of 5000 cells per well. After treatment with or without ShhN CM supplemented with various concentrations of solasoninefor 72 h. The alkaline phosphatase activity was measured using a kit from Beyotime on a plate reader (Molecular Devices) at 405 nm.

### 4.4. Quantitative RT-PCR (RT-qPCR)

Total RNA was extracted from cells or medullbolbatoma tissues using Trizol reagent (Takara, Dalian, China) following the manufacturer’s protocol. The reverse transcription of RNA and qPCR were performed using the following primers:

*mGUSB*: Forward-5’CTGCCACGGCGATGGA3’

Reverse-5’ACTGCATAATAATGGGCACTGTTG3’

*mGli1*: Forward-5’GCAGTGGGTAACATGAGTGTCT3’

Rreverse-5’AGGCACTAGAGTTGAGGAATTGT3’

*mPtch1*: Forward: 5’-GCTACGACTATGTCTCTCACATCAACT-3’

Reverse: 5’-GGCGACACTTTGATGAACCA-3’

The mRNA levels of interested genes were normalized to that of GUSB.

### 4.5. Lentivirus

The lentiviral stocks were prepared according to routine protocol. Briefly, the plasmid carrying the Sufu-shRNA and three packaging plasmids were co-transfected into HEK293T cells using Lipofectamine 2000 (Invitrogen, Grand Island, NY, USA). The viruses were harvested 24 h post transfection and used for infection of NIH-3T3 cells seeded in 10-cm dishes. Cells were collected for testing the expression of Sufu at 5–7 days after infection by western blot analyses.

### 4.6. Western Blot Analysis

Cells after various transfections as indicated were collected and subjected to lysis buffer (50 mM Tris, pH 7.4, 150 mM NaCl, 1% NP-40, 1 mM sodium vanadate, 1 mM PMSF, 1 mM DTT, 10 mg/mL of leupeptin and aprotinin), followed by immunoblot analysis. Primary antibodies against Sufu, Smo, and GAPDH (Santa Cruz Biotechnology, Santa Cruz, CA, USA) were used for immunoblot analysis according to the routine procedure.
